# A Novel CNN-Based CAD System for Early Assessment of Transplanted Kidney Dysfunction

**DOI:** 10.1038/s41598-019-42431-3

**Published:** 2019-04-11

**Authors:** Hisham Abdeltawab, Mohamed Shehata, Ahmed Shalaby, Fahmi Khalifa, Ali Mahmoud, Mohamed Abou El-Ghar, Amy C. Dwyer, Mohammed Ghazal, Hassan Hajjdiab, Robert Keynton, Ayman El-Baz

**Affiliations:** 1 Bioengineering Department, University of Louisville, Louisville, KY USA; 2 Radiology Department, Urology and Nephrology Center, Mansoura University, Mansoura, Egypt; 3 Kidney Disease Program, University of Louisville, Louisville, KY USA; 4 Electrical and Computer Engineering Department, Abu Dhabi University, Abu Dhabi, UAE

## Abstract

This paper introduces a deep-learning based computer-aided diagnostic (CAD) system for the early detection of acute renal transplant rejection. For noninvasive detection of kidney rejection at an early stage, the proposed CAD system is based on the fusion of both imaging markers and clinical biomarkers. The former are derived from diffusion-weighted magnetic resonance imaging (DW-MRI) by estimating the apparent diffusion coefficients (ADC) representing the perfusion of the blood and the diffusion of the water inside the transplanted kidney. The clinical biomarkers, namely: creatinine clearance (CrCl) and serum plasma creatinine (SPCr), are integrated into the proposed CAD system as kidney functionality indexes to enhance its diagnostic performance. The ADC maps are estimated for a user-defined region of interest (ROI) that encompasses the whole kidney. The estimated ADCs are fused with the clinical biomarkers and the fused data is then used as an input to train and test a convolutional neural network (CNN) based classifier. The CAD system is tested on DW-MRI scans collected from 56 subjects from geographically diverse populations and different scanner types/image collection protocols. The overall accuracy of the proposed system is 92.9% with 93.3% sensitivity and 92.3% specificity in distinguishing non-rejected kidney transplants from rejected ones. These results demonstrate the potential of the proposed system for a reliable non-invasive diagnosis of renal transplant status for any DW-MRI scans, regardless of the geographical differences and/or imaging protocol.

## Introduction

Chronic kidney disease (CKD) is the gradual loss of the kidney’s ability to remove waste and excess fluids from blood. In the U.S., approximately 30 million patients have CKD^1^, which if it remains untreated, will result in progressive damage of the kidney until it develops a fatal condition called end stage renal disease (ESRD). In 2014, the estimated number of ESRD patients in the U.S. was 780,000^1^. ESRD is treated by blood dialysis and eventually by kidney transplant. While dialysis helps the patient stay alive, it performs only 10% of the kidney’s function which leads to dangerous health conditions. Meanwhile, transplantation is considered a long-term treatment as it prolongs patients’ lives. However, organ procurement and transplantation is a challenging process. Each month, more than 3,000 patients are added to the kidney transplant waiting list^2^. During the first 5 years after transplantation, there is a 15% chance that the immune system will reject the foreign organ, leading to kidney dysfunction^3^. Therefore, the salvation of the transplanted kidney is of a great medical importance. The types of renal rejection include: hyper-acute, acute, and chronic. Acute rejection (AR) is the leading cause of graft dysfunction^4^, and is therefore the focus of this study. Measuring the glomerular filtration rate (GFR) after renal transplantation has been accepted by the National Kidney Foundation as a diagnostic methodology for assessing renal graft function^5^. However, this method is a late indicator and suffers from low sensitivity. Renal biopsy is the gold standard for graft function evaluation and can determine the root cause and the type of graft dysfunction^4^. However, surgical biopsies are expensive and are invasive methods that carry a risk for serious complications, such as bleeding and kidney injury. Moreover, it can underestimate the severity of the problem due to the fact that it only tests a small portion of the kidney^6^.

Medical imaging modalities have provided an accurate, non-invasive way to detect AR and its underlying cause. For example, radionuclide imaging is successful in assessing graft function due to its ability to capture tissue function^7^. Nevertheless, the exposure to radiation is associated with health risks. The emerging technique of helical computed tomography (CT) that uses less nephrotoxic contrast has been proven successful in the diagnosis of post-transplant complications^8^. Although this technique is safer than radionuclide imaging, it has a low specificity, and the usage of contrast agents still adds some nephrotoxicity. Ultrasound (US) imaging is used to detect allograft dysfunction and it is better than other imaging techniques in terms of cost, ease of use, and health risks. However, this method has shadowing artifacts and low signal-to-noise ratio, and there has been a debate about the validity of the measures used in this method^9,10^.

A variety of advanced magnetic resonance imaging (MRI) techniques have gained considerable attention in kidney transplant function assessment or diagnosing structural kidney disease due to superior soft tissue contrast. For example, dynamic contrast enhanced (DCE) MRI uses contrast agents such as gadolinium to evaluate tissue perfusion, which is indicative of renal function^11^. While DCE-MRI provides good anatomical and functional information, gadolinium may adversely affect the kidney and cause nephrogenic systemic fibrosis^12^ when GFR < 30 ml/min per 1.73 m^2^. Blood oxygen level dependent (BOLD) MRI that evaluates renal oxygenation status has also been used in the detection of AR^13^. However, BOLD-MRI has several limitations such as bowel gas artifacts and susceptibility to breathing motion artifacts^14,15^.

Diffusion weighted (DW) MRI, which is an imaging sequence that does not use contrast agents, has been sucessful in many applications, such as tumor detection and characterization, neuroimaging, and kidney function assessment^16^. It measures the motion of water molecules inside the tissue, and thus helps to assess the diffusion characteristics of the tissue. Quantitative maps, known as apparent diffusion coefficient (ADC), that represent the diffusion can be obtained at different magnetic field strengths and duration (b-value)^16^. The feasibility of using DW-MRI in assessing kidney allograft function has been investigated in several studies^17–27^. The ADC was utilized as a discriminatory feature to differentiate between biopsy proven stable renal allograft and pathological allografts with issues such as AR or acute tubular necrosis (ATN). The results in those studies showed that normal functioning kidneys had higher ADC values compared to kidneys with deteriorated function. Also, most of the studies showed a positive correlation between the ADC values and the estimated GFR. However, those studies have multiple shortcomings. First, they estimated the average ADCs from the middle or the largest cross-section at certain b-values only. Second, they did not investigate the effect of integrating the imaging and clinical biomarkers to differentiate between stable and AR allografts. Finally, the benefit of applying advanced machine learning (ML) techniques was not utilized as a valuable tool for image classification and diagnosis.

To partially overcome those limitations, recent studies have utilized various ML techniques to improve the diagnostic accuracy of the CAD systems by extracting more learnable features from the underlying data. ML has shown significant success in many diverse medical imaging applications, such as pattern classification, anomaly detection, and image segmentation and registration^28^. Thus, ML methods are indispensable tools in modern CAD systems that assist in making decisions regarding medical diagnosis. Traditional ML approaches depend heavily on feature engineering to convert the data into suitable patterns, which is a tedious task as it requires time and field domain expertise to determine the best features to extract^29^. Recently, deep learning (DL) has evolved as an exciting field, inspired by artificial neural networks (ANN) in which the network has many hidden layers^29^. This technique builds a hierarchical data representation (i.e. from less to more abstracted representations), and thus has the power of learning high-level features from the underlying data and avoids the burden of extracting the relevant features^30^.

In literature, there has been limited work in the area of applying CNN for kidney diagnosis and classification purposes. Kidney diseases can be diagnosed by detecting and evaluating the tissue regions corresponding to glomeruli. Pedraza *et al*.^31^ proposed a CNN model that is based on a pre-trained AlexNet^32^ to classify between glomerular and non-glomerular tissue. Their network was fed by images from tissue slides which were adapted from kidney biopsies, and it subsequently achieved a performance of 0.999 (F-score). Also, the glomerulus detection accuracy of CNNs fed with whole-slide kidney images surpassed the histogram of oriented gradients classifiers^33^. Yang *et al*.^34^ used CNNs to classify the histological kidney images generated from tissue microarrays that have been obtained using biopsies from tumors and normal cases. They achieved a high classification accuracy of 97– 98%. Kolachalama *et al*.^35^ investigated the potential of CNNs in the prediction of the CKD stage, baseline serum creatinine, and nephrotic-range proteinuria from the processing of trichrome-stained images generated from renal biopsy samples. They reported that the proposed CNN had the same performance as an expert nephropathologist. Most recently, March *et al*.^36^ extended a pre-trained CNN model to classify between non-sclerosed and sclerosed glomeruli in frozen sections adapted from kidney biopsies. The goal of their work was to evaluate the eligibility of donated kidneys prior to transplantation to minimize post-transplantation complications. Despite the success of the aforementioned studies^31,33–36^ in building computational models that assess kidney function, they are predicted upon an invasive procedure, i.e. biopsy.

**Figure 1:**
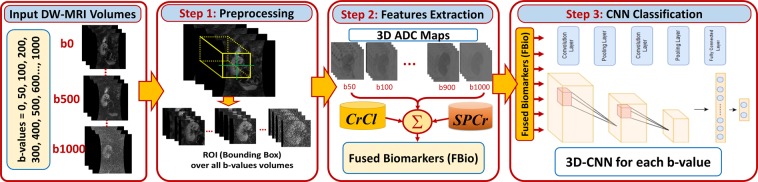
The proposed convolutional neural network (CNN)-based framework for early detection of renal transplant rejection using diffusion-weighted (DW) MRI. This system consists of three main processing steps. In the first step, the histogram of input DW-MRI data is equalized to reduce the noise and inhomogeneity of intensities. Then, an ROI enclosing the kidney in each subject is constructed. In the second step, the 3D ADC maps are estimated for the selected ROI and then fused with the clinical biomarkers, i.e., the creatinine clearance (CrCl) and serum plasma creatinine (SPCr), for allografts classification. In the final step, the fused markers are fed as a 3D input of size 150 × 150 × 24 voxels to the proposed CNN-based classifier to classify renal allografts into non-rejection (NR) and acute rejection (AR).

To overcome those shortcomings, we propose an automated framework, shown in Fig. 1, that combines the advantages of both DW-MRIs and DL to classify renal allografts into non-rejection (NR) and AR status by using data generated from the fusion of voxel-wise ADCs and the clinical biomarkers. To the best of our knowledge, this is the first automated non-invasive CAD system of its kind to assess renal transplant status using the integration of the DW-MR image markers and clinical biomarkers along with CNN.

## Materials and Methods

In this study, a DW-MRI dataset of 56 individuals (with associated clinical biomarkers), who had renal transplantation, was used for the evaluation of the proposed CAD system. All experimental protocols were approved by by the Institutional Review Boards (IRB) of the University of Michigan, USA; University of Louisville, USA; and University of Mansoura, Egypt. The methods were carried out in accordance with the relevant guidelines and regulations. All participants and/or their legal guardians were fully informed about the aims of the study and provided their written and/or verbal consent. Thirty-eight of the participants were males and 18 were females, and they had an average age of 34 ± 15.62 with a range of 12–65. These subjects were divided into two groups: individuals with stable graft functionality or the NR group (N = 26), and participants who had been diagnosed with graft dysfunction or the AR group (N = 30).

Seventeen of the DW-MRI scans in this dataset were acquired in the USA using a 3T scanner: MRI Ingenia, Philips Medical System, Amsterdam, Netherlands; a body coil and a gradient single-shot spin-echo echoplanar sequence; TR/TE: 8000/93.7; slice size: 256 × 256 pixels; section thickness: 4 mm; inter-section gap: 0 mm; FOV: 360 × 360 × 152 mm^3^. A total of 38 coronal cross-sections were obtained for a total acquisition time of 60 s to cover the whole kidney. In dditiona, 5 scans were acquired in Egypt using a 3 T scanner with the following characteristics: MRI Ingenia, Philips Medical System, Amsterdam, Netherlands using a body coil and a gradient single-shot spin-echo echoplanar sequence; TR/TE: 4400/82; slice size: 176 × 176 pixels; section thickness: 4 mm; intersection gap: 0 mm; FOV: 220 × 195 × 96 mm^3^. A total of 24 coronal cross-sections were obtained at each b-value for an acquisition time of 30– 60 s to cover the whole kidney. In addition, 34 scans were acquired in Egypt using a 1.5T scanner: SIGNA Horizon, General Electric Medical Systems, Milwaukee, WI using a body coil and a gradient single shot spin-echo echoplanar sequence; TR/TE: 8000/61.2; slice size: 256 × 256 pixels; section thickness: 4 mm; intersection gap: 0 mm; FOV: 360 × 360 × 152 mm^3^. Approximately 50 coronal cross-sections sections were obtained in 60–120 s to cover the entire kidney. The DW-MRIs, clinical biomarkers, and biopsies were included in the final analysis and examined by a nephrologist and a radiologist.

From a clinical point of view, the renal allograft motion is not challenging because the allograft is implanted in the iliac region, and thus, a transplanted kidney is less affected by respiration than a native kidney. Moreover, patients were asked to hold respiration (breath) during the study to reduce possible respiratory motion effects. Therefore, in this specific application, we not only believe that a single direction will lead to an accurate estimation of the ADCs, but also will reduce the acquisition time (≈12 min for the single-direction compared to ≈31 min for the three-direction). This in turn provides the ability for acquiring a 12 b-value scan (lower b-values capture the blood perfusion effect and higher b-values capture the water diffusion effect), which will provide a more accurate final diagnostic performance (final decision is based on the integration of the decisions from all of the individual b-values).

In this study, every sequence of the aforementioned sequences is a single DW-MRI direction noted by direction cosines “X, Y, Z = [1, 1, 1]”. That is, all three gradient channels were run simultaneously at equal amplitude. This sequence acquired b-values = 0, 50, 100, 200, 300, 400, 500, 600, 700, 800, 900, and 1000 s mm^−2^.

A fully automated CAD system based on a CNN classifier for the early detection of acute renal transplant rejection was developed in this study. To obtain the final diagnostic results as NR or AR renal allografts, three main processing steps are performed: (i) data prepossessing and ROI selection; (ii) extraction/estimation of 3D ADC maps for the selected ROI and its fusion with the clinical biomarkers (i.e., the CrCl and SPCr) to be used as discriminatory features for allografts classification; and (iii) classification of transplant status by using the fused markers as input to a CNN-based classifier. Details of these steps are presented in the following sections.

### Data Preprocessing and Region-of-Interest (ROI) Selection

The DW-MRI data were first preprocessed by performing a histogram equalization (i.e. intensity normalization) with a non-parametric bias field correction^37^ in order to reduce the noise and inconsistencies due to low-frequency non-uniformity, or inhomogeneity of intensities. Following preprocessing, an ROI enclosing the kidney was constructed in each subject. The size of the ROI was determined based on the largest kidney volume in our database and was held constant for all subjects. Then, the slice containing the largest cross-section of the kidney was selected, and a software user identified the approximate centroid of the cross-section in order to localize a rectangular ROI that encompasses the whole kidney. For each subject, the DW-MRI kidney volumes were cropped at all b-values based on this rectangular ROI. This step essentially has two advantages. First, it reduces the complexity of the proposed pipeline by avoiding complex segmentation and registration methods. Second, this also has the advantage of reducing the time needed for the classification steps, as the volumes used for training and testing are largely reduced. In our experiment below, the original kidney volumes range in size from 176 × 176 × 24 to 256 × 256 × 38 voxels, while the cropped ROI-kidney volume used was 150 × 150 × 24 voxels.

### ADC Maps

The diffusion, or Brownian motion of water molecules in the human tissue, depends on its micro-structure and function. Therefore, the presence of an abnormality in the tissues is characterized by a change in the normal diffusion pattern^16^. Thus, the evaluation of the kidney diffusion pattern gives us a quantitative insight into the diffusion characteristics of that tissue (i.e. renal tissue) and insight about its current state and potential pathologies^18^. The contrast in a DW image is created from the variations in random motion of water molecules within the tissue. In a given DW-MRI sequence, the recorded signal is attenuated in accordance with the Stejskal-Tanner equation^16^:

1 $${S}_{b}={S}_{0}{e}^{-b\cdot {\rm{ADC}}}$$

where *b*, *S*_*b*_, *S*_0_, and ADC are the diffusion gradient strength and duration (b-value), signal intensity at *b*, baseline signal intensity (*b* = 0 s mm^−2^), and apparent diffusion coefficient. The b-value is determined by^16^:

2 $$b={\gamma }^{2}{G}^{2}{\delta }^{2}({\rm{\Delta }}-\frac{\delta }{3});$$

**Figure 2:**
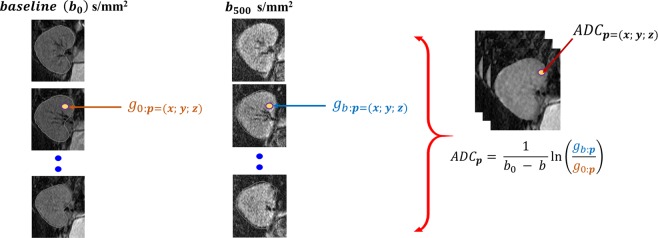
Illustration of the voxel-wise ADC calculations at a voxel (x; y; z) at b-value of 500 s mm^−2^: (**a**) the ROI-kidney regions at *b*_0_, (**b**) the ROI-kidney regions at *b*_500_, and (**c**) the constructed ADC maps for the defined ROI.

*γ* is the gyro-magnetic ratio of hydrogen, equal to 42.58 MHz/T; *G* and *δ* are the magnitude and duration of the gradient magnetic field; Δ is the time interval between gradient pulses; and *S*_0_ is the signal intensity in the absence of a gradient. It can be drawn from Eq. (1) that the higher the b-value, the more signal attenuation. An ADC map, illustrated in Fig. 2, that quantifies the diffusion at a specific b-value, can be obtained from two DW images, one at b = 0 and the other at that specific b-value by:

3 $${\rm{ADC}}=\frac{-(\mathrm{ln}({S}_{b})-\,\mathrm{ln}({S}_{0}))}{b}$$

ADC maps have several advantages over raw DW images: (1) the effects of *T*_1_ and *T*_2_ contrast and *T*_2_ shine through are removed; (2) ADC maps represent only the diffusion inside the tissues; and (3) the ADC values are directly proportional to diffusion. Since the AR group has lower ADC values^18^, the estimation of ADC maps for renal allografts gives more insight into its functional state and will help us differentiate between the NR and the AR groups.

### Clinical Biomarkers

Different biomarkers of kidney function are used in clinical practice. These markers include creatinine, which is a metabolic waste product of creatine in muscle. SPCr and CrCl tests measure the level of creatine in the patient’s blood and urine, respectively. Therefore, these tests can be used as indicators for kidney function and GFR. The SPCr and CrCl laboratory values are usually measured for patients after the transplantation procedure^4^.

### Data Preparation: ADCs and Biomarkers Fusion

**Figure 3:**
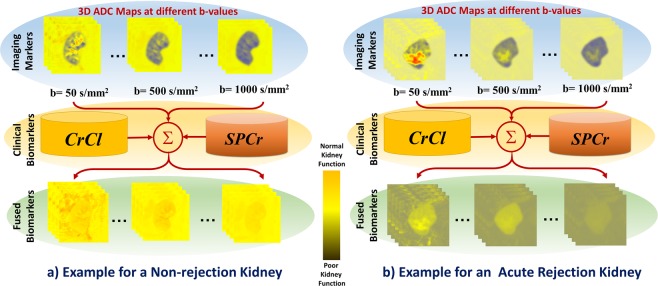
Demonstration of the efficacy of the fusion of both image and clinical markers. In this work, the estimated ADC maps are fused with the one dimensional (1D) CrCl and 1D SPCr values obtained during routine post-transplantation monitoring. The clinical biomarkers of each subject were first normalized with regard to the maximum values of each marker. Then, the normalized values were added to the voxel-wise ADC maps at all of the b-value scans. This difficult example differentiates an NR case (**a**) from an AR case (**b**). As illustrated in this figure, it is very difficult using the ADC maps alone to distinguish between the normal and abnormal subjects. This can be justified by the large overlap of the ADC values between these two subjects, which have been revealed by the color-coded maps. Visually, it is clear that the two subjects had a good color separation after the fusion process of both markers, where the dark-green color represents poor kidney function (i.e. low ADCs + low CrCl + high SPCr) and the orange-yellowish color represents a normal kidney function (i.e. high ADCs + high CrCl + low SPCr).

In order to train our ML model with data that have high discriminatory power, the estimated ADC maps were fused with the CrCl and SPCr values obtained during routine post-transplantation monitoring. The clinical biomarkers of each subject were added to the ADC maps at all of the b-value scans as shown in Fig. 3, and the fused data are abbreviated as FBio. To highlight the advantages of the fusion process of both markers, two main scenarios were employed. First, the constructed ADC maps were used alone to assess the functionality of the allograft. Second, clinical biomarkers were fused with the constructed ADC maps to build the FBio maps, which in turn were used to assess the functionality of the allograft. In both scenarios, these maps were constructed at the 11 different b-values from the available 56 subjects (26 NR and 30 AR). Then, both types of maps were input to train and test a CNN-based classifier using a leave-one-subject-out (LOSO) cross-validation approach. Details about the proposed CNN-based classifier are discussed in the following section.

### Convolutional Neural Networks (CNN)

**Figure 4:**
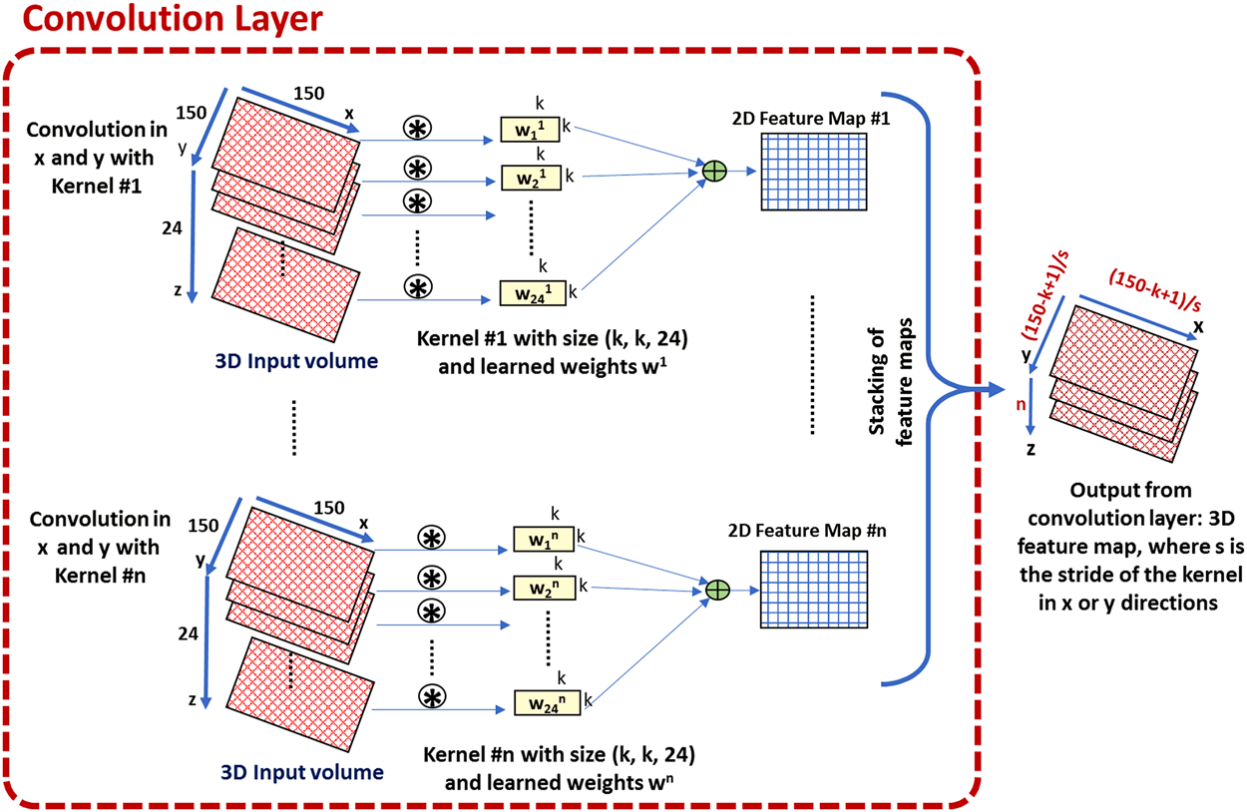
Illustration of the processing of a single volume using CNN. To clarify how a single convolution layer of the CNN processes a 3D input volume of size 150 × 150 × 24 voxels, each 3D volume has 24 images and a 2D convolution is applied to each image using a kernel of size *K* × *K*. The output of an individual kernel is then summed to create a 2D feature map. Usually, multiple kernels are used, and the above step is applied again for those kernels, and different feature maps are produced for respective kernels. The final result is a volume of feature maps.

CNN is a machine learning model that is inspired by deep ANN^38^. CNNs have gained a lot of attention in medical image analysis due to their ability to maintain spatial relationships of processed images, which gives them an advantage over the traditional ANNs^30^. The basic building units of the CNN are convolution layers, non-linearity layers, pooling layers, and fully connected (FC) layers. The first layer is the convolution layer in which the input image or volume is convolved with a set of learned filters, as illustrated in Fig. 4. The output of the convolution layer is a set of feature maps equal in number to the filters. Each map represents the places of strong activations associated with a specific filter. The convolution operation between an image *I* of size *M* × *N* and a filter *W* of size *A* × *B* that results in a feature map *s* is defined by the following dot product:

4 $$s=\sum _{a}\,\sum _{b}\,I(a,b).W(m-a,n-b)$$

The non-linearity layer accounts for the interaction effects between the factors that influence the prediction. Also, it introduces a non-linearity into the model because it is expected that the real-world problems are non-linear. In our CNN, we used the popular rectifying linear unit (ReLU)^32^ that operates element-wise on the feature map to produce an activation map as follows:

5 $$f(x)=\,{\max }(0,x)$$

where *x* refers to an input to a neuron. The advantages of using ReLU over traditional activation functions (logistic and hyperbolic tangent functions) are that it reduces the required training time by avoiding the “vanishing gradient problem”. The pooling layer reduces the spatial resolution of the activation map and keeps the spatial relationships among the features of high activation. Therefore, it decreases the computational cost by decreasing the number of parameters and produces abstract representation that helps avoid over-fitting. Pooling types include max-pooling and average-pooling. The max-pooling replaces the pixels inside a window with the maximum value whereas the average-pooling replaces the pixels with the average value. The FC layers are the final layers in a CNN, and there can be one layer or many layers. The phrase fully connected means that every neuron in a previous layer is connected to all neurons in the next layer as with the traditional ANN. Tuning the CNN’s parameters to a particular problem is a quite challenging task because there are many hyper-parameters related to the network architecture and training. The following section discusses our procedure to tune our CNN parameters.

#### The Proposed CNN Architecture

**Figure 5:**
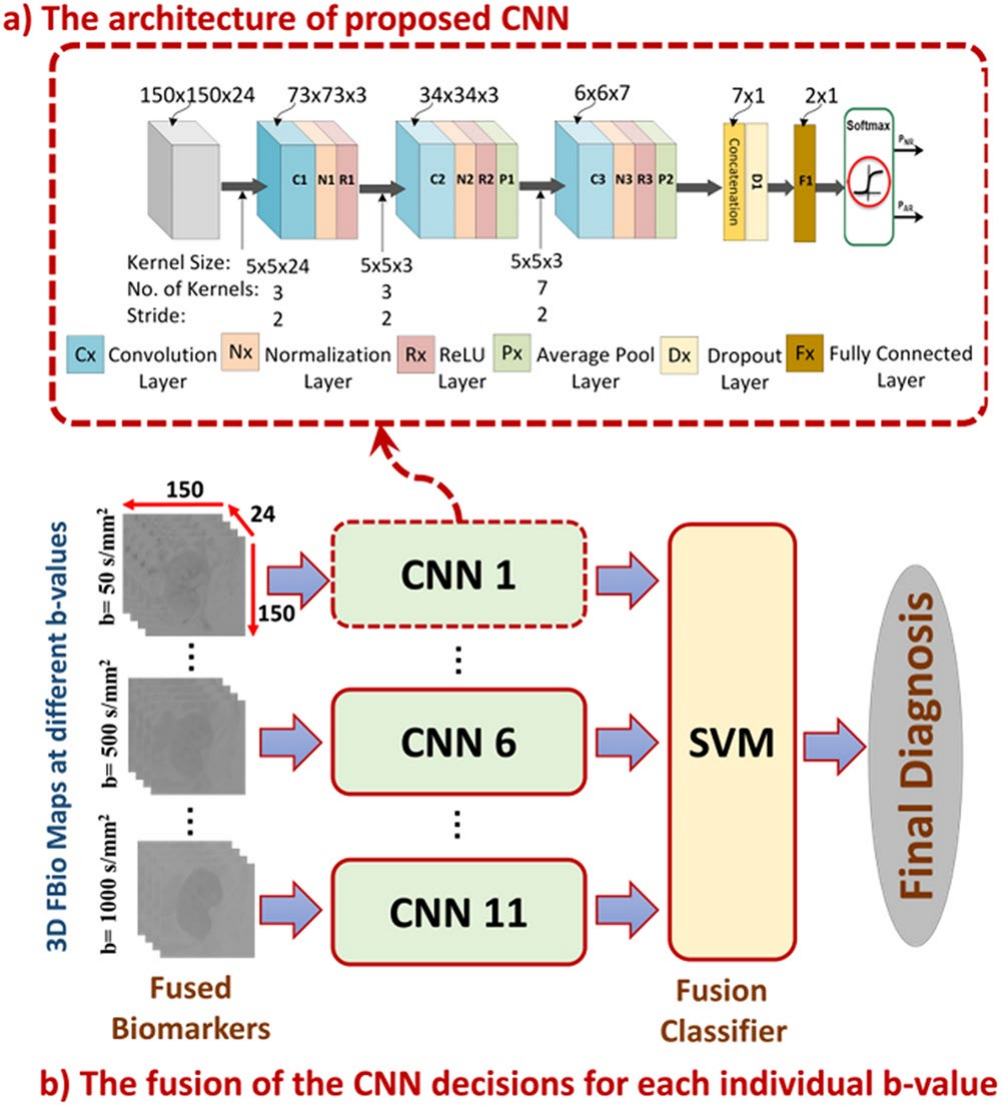
The architecture of the proposed convolutional neural network (CNN), where (**a**) illustrates the configurations of the developed CNN and its layers and (**b**) represents the pipeline of the fusion process using a support vector machine (SVM) classifier. The CNN is trained and validated at each b-value apart from the other b-values. At each b-value, we have 56 samples. Each sample is a 3D volume for a certain subject, and each volume is 150 × 150 × 24 voxels. Each 3D volume is fed to a CNN as one sample of the dataset. The final classification decision is obtained by combining the decisions of all CNNs’ output probabilities at all b-values. This fusion is achieved by a support vector machine (SVM) where each sample has 22 features (2 probabilities for the two classes obtained from the CNN at a specific b-value × 11 b-values).

To find the best CNN architecture, a total of 20 subjects (10 AR and 10 NR) were only used as an independent training dataset and were not used during framework validation. A grid search method was employed to search for the number of convolution layers (range: 2–5), number of neurons in the linear layer (range: 3–15), number of convolution kernels (range: 3–15), convolution or pooling kernel size (range: 3–7), and kernel stride (range: 1–3) with the classification accuracy as the score to be optimized until we got an average global accuracy of 96.4% from ten training iterations in row. We organized our network as processing blocks, where each block starts with a convolution layer followed by a batch-normalization layer to accelerate the network training^39^, followed by a ReLU activation layer, see Fig. 5(a). At the end of each block, there might be an average pooling layer based on the grid search result. Our proposed network has three processing blocks followed by concatenation and an FC layer, as shown in Fig. 5(a). The input to the first block is the 3D fused data with the size of 150 × 150 × 24 voxels. The output of the third block is concatenated to form a vector of seven neurons and then fed into an FC layer of two neurons for the two classes. Finally, the output from the FC layer is fed into a soft-max classifier to compute a posteriori class probability. For more information about the network configuration, see Fig. 5(a) and Table 1.

**Table 1:** The proposed CNN configuration.

Layer	Depth	kernel	Stride	Spatial Size	Parameters
Input	24	—	—	150 × 150 × 24	0
1. Conv.	3	5 × 5	2 × 2	73 × 73 × 3	5 × 5 × 24 × 3
2. Conv.	3	5 × 5	2 × 2	34 × 34 × 3	5 × 5 × 3 × 3
3. Avg	3	5 × 5	2 × 2	15 × 15 × 3	0
4. Conv.	7	5 × 5	2 × 2	6 × 6 × 7	5 × 5 × 3 × 7
5. Avg	7	6 × 6	1 × 1	1 × 1 × 7	0
6. Concat.	1	—	—	7 × 1	0
7. Full	1	—	—	2 × 1	7 × 2
Total number of parameters = 2564					

#### CNN Training and Testing

The stochastic gradient descent (SGD) was used to train and test our network. The learning rate was set to 0.1 and then was reduced by a factor of 10 to reach 0.0001 over 70 epochs. The complex architecture of deep neural networks and convolutional networks makes them susceptible to the problem of overfitting, in which the network learns features peculiar to the training dataset and fails to generalize, i.e. to correctly analyze novel input. Dropout is one of the most efficient techniques to override overfitting^40^. In dropout, the relationships found in the data can be modeled using various representations. This is achieved by randomly deactivating a proportion of neurons in each iteration during training. By dropping the neurons, we prevent complex co-adaptations during training. Although the dropout technique deactivates some neurons randomly in each iteration, these neurons might be active in the next iteration. In our model, we used a dropout factor of 0.5. Our network was implemented using PyTorch deep learning framework^41^, and all of the computations were performed on an NVIDIA Quadro P4000 GPU.

#### CNN Decision Fusion

The proposed CNN was used to process FBio maps at 11 different b-values. Then, the predicted probabilities assigned for each class were collected for each subject at the 11 individual b-values. To obtain a better classification accuracy, we fused all of the 11 decisions of the individual CNNs by using a support vector machine classifier (SVM). The 22 probabilities (2 classes × 11 b-values) for each subject were fed to an SVM classifier with a linear kernel to obtain the final classification.

## Experimental Results

The proposed pipeline was evaluated on the above-mentioned 56 samples using LOSO and 10-fold cross-validation approaches. In this study, the CrCl values for the NR group averaged 72.62 ± 17.58 ml/min, and the SPCr averaged 1.16 ± 0.23 mg/dl. In the AR group, CrCl values averaged 39.63 ± 11.98 ml/min and the SPCr averaged 2.23 ± 0.71 mg/dl. Demonstration of the efficacy of the fusion of both image and clinical markers is illustrated in Fig. 3, which differentiates an NR case and an AR case. As demonstrated in Fig. 3, it is very difficult using the ADC maps alone, shown in Table 2, to distinguish between the normal and the abnormal subjects. This can be justified by the large overlap of the ADC values between the two subjects, which have been easily revealed by the color-coded maps. To show the effect of fusing the clinical biomarkers with the imaging markers, the FBio were then used as our new input discriminatory features for the renal transplant status assessment. Visually, it is clear that the two subjects had a good color separation after the fusion process.

**Table 2:** A comparison example between a non-rejection (NR) and an acute rejection (AR) subjects based upon the mean and the standard deviation (std) values of the ADC maps alone at the 11 individual b-values.

ADC Maps at Individual b-values: mean(std)≈											
b-value	b50	b100	b200	b300	b400	b500	b600	b700	b800	b900	b1000
NR	3.0 (1.17)	2.63 (0.72)	2.59 (0.50)	2.46 (0.41)	2.43 (0.37)	2.21 (0.33)	2.10 (0.08)	2.09 (0.26)	2.05 (0.25)	1.95 (0.23)	1.90 (0.21)
AR	2.85 (1.79)	2.97 (0.98)	2.81 (0.53)	2.33 (0.42)	2.28 (0.29)	2.12 (0.25)	2.09 (0.24)	1.93 (0.22)	1.90 (0.22)	1.87 (0.19)	1.83 (0.17)

In addition to the fusion of clinical and image biomarkers, the integration of the diagnostic results obtained from individual b-values is expected to enhance the final global diagnostic accuracy. As described in Section 2.4, training and testing of the CNN-based classifier were performed using the ADC maps only or the FBio result from the fusion process. For both scenarios, these maps were constructed at the 11 different b-values for all available subjects. The individual accuracies at each b-value and the average global accuracy for both scenarios are reported and compared in Table 3. To confirm the robustness of the proposed approach, a 10-fold cross-validation scenario was performed on the same data using the FBio and the same CNN-based classifier, with resulting accuracy of 91%, sensitivity of 90%, and specificity of 92%. To determine the contribution of imaging vs. clinical biomarkers to overall diagnostic accuracy, the performance of the CNN using FBio was compared with that of using ADC maps alone, as well as that of a support vector machine (SVM) classifier using the clinical biomarkers alone, abbreviated as ClinBio, (i.e. CrCl and SPCr). The results are summarized in Table 4 in terms of accuracy, sensitivity, and specificity.

**Table 3:** Comparison of the approximated percentage diagnostic accuracy (ACC%) obtained at individual and fused b-values (F_11_) between the first scenario (*S*_1_) using the ADC maps alone with the CNN-based classification system, the second scenario (*S*_2_) utilizing the fusion of the clinical biomarkers and the image markers using the same CNN-based classification system, and the classification using the CDFs of the ADCs obtained from the segmented kidney based upon use of stacked auto-encoders (SAEs)^42^.

Individual b-values Classification Accuracy (ACC%)≈												
Approach	b50	b100	b200	b300	b400	b500	b600	b700	b800	b900	b1000	F_11_
*SAEs* (CDFs)^42^	68	50	**77**	68	82	68	73	64	75	86	68	86
*S*_1_ (ADC only)	59	50	64	59	59	59	68	64	77	73	68	82
*S*_2_ (FBio)	68	**68**	73	**80**	**83**	**73**	**85**	**86**	75	86	**86**	**93**

**Table 4:** Comparative diagnostic quality between the first scenario (*S*_1_) using the ADC maps alone with the CNN-based classification system, the second scenario (*S*_2_) utilizing the fusion of the clinical biomarkers and the image markers using the same CNN-based classification system as well, the clinical biomarkers (ClinBio) based on using a support vector machine (SVM) classifier, and the classification using the CDFs of the ADCs obtained from the segmented kidney based on using stacked auto-encoders (SAEs)^42^ in terms of accuracy (ACC%), sensitivity (SENS%), specificity (SPEC%), and area under the curve (AUC) for the combined/fused decision from all of the individual b-values.

Quality of the Final Diagnosis				
Approach	ACC%≈	SENS%≈	SPEC%≈	AUC≈
*SAEs* (CDFs)^42^	86	70	100	0.88
*S*_1_ (ADC only)	82	80	83	0.83
*S*_2_ (FBio)	93	93	92	0.93
*SVM* (ClinBio)	77	80	73	0.80

In addition to the fusion of clinical and image-derived biomarkers, the overall accuracy of the proposed framework is also highlighted by comparing its performance to other learning approaches. Namely, we compared its performance against the autoencoding-based technique using stacked auto-encoders (SAEs) utilizing the cumulative distribution functions (CDFs) of the ADC maps estimated from segmented kidney objects^42^. The comparison results at all b-values are summarized in Table 3. Additionally, the overall accuracy, sensitivity, and specificity of the proposed pipeline and SAEs(CDFs) technique^42^ are compared in Table 4. As clearly shown in the reported results, the proposed CNN-based system outperforms the SAEs(CDFs) method^42^. The advantage of the proposed method over the SAEs(CDFs) method^42^ is that our new renal rejection diagnostic system requires neither alignment nor kidney segmentation as has been done previously^42^, which might be challenging in some cases due to diffused boundaries and inter-patient anatomical differences; therefore, the presented renal rejection CAD system will have reduced computational time and complexity.

**Figure 6:**
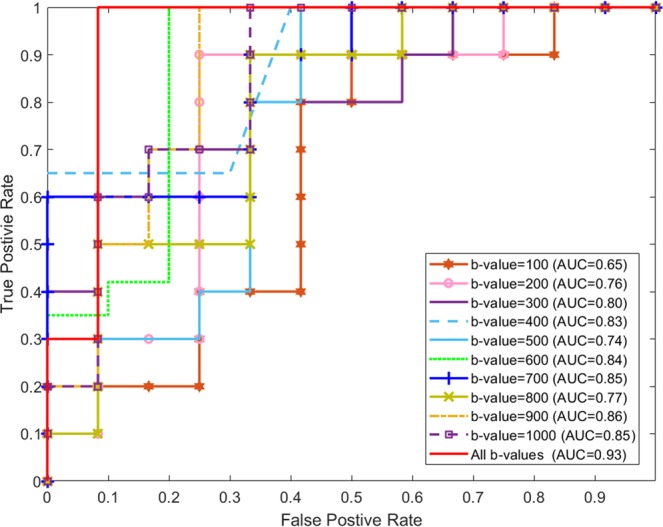
Receiver operating characteristics (ROC) curves for the FBio scenario for individual b-values and their fusion.

**Figure 7:**
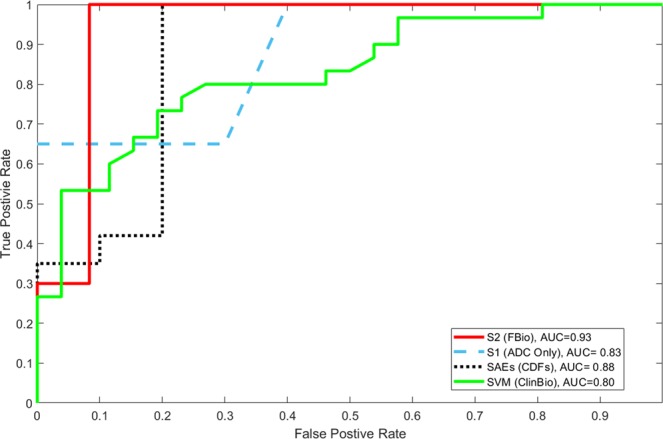
Receiver operating characteristics (ROC) curves for the proposed CNN-based system (for both scenarios *S*_1_ and *S*_2_), the clinical biomarkers (ClinBio) based upon using a support vector machine (SVM) classifier, and the auto-encoding system^42^.

Furthermore, robustness of our CAD system has also been analyzed using the receiver operating characteristic (ROC). Figure 6 demonstrates the ROC curves for individual b-values as well as their fusion using the proposed pipeline. The areas under the curve (AUC) for individual b-values are shown. As discussed earlier, the fusion of the b-values enhanced the overall accuracy, which is also documented by the AUC = 0.93. Additionally, a comparison between SAEs(CDFs) method^42^ and all scenarios studied in this paper using the ROC are shown in Fig. 7. In particular, the SAEs(CDFs) method^42^ achieved an AUC 0.05 less than the CNN method described here. The above evaluation and comparison results demonstrated the higher accuracy and robustness of our developed framework for the detection of AR.

## Discussion and Conclusions

Early detection of AR can help physicians with early intervention with appropriate treatment and thus prolong the renal graft function and improve patient outcomes. Generally, there are multiple types of AR, and the selection of the appropriate treatment depends on the rejection type. For example, acute cellular rejection is treated with a high dose of corticosteroids, administrated intravenously as the first line treatment^43,44^. The most popular regimen is the administration of methylprednisolone for three successive days^43^. In the case of persistent kidney deficiency with the steroid and/or antithymocyte globulin or the presence of a new defect in renal function after treatment of AR, another biopsy is recommended to discover additional causes of renal dysfunction. T-cell depleting antibodies are suggested for aggressive vascular cellular rejection and AR episodes that do not respond to steroid treatments^45^. On the other hand, if antibody mediated rejection is the resulting diagnosis, the following alternatives are suggested for treatment: plasmapheresis, immunoadsorption, intravenous immunoglobulin, or monoclonal antibodies^46^.

It is worth mentioning that most of the clinical research estimates the ADC at a few select b-values^17–27^, typically one of the lower b-values and one of the higher b-values along with the baseline (b0). In fact, blood flow and complex tissue microstructure create non-monoexponential DW-MRI signal attenuation. The maximal b-value in this study (1000 s mm^−2^) should not elicit excessive non-Gaussian diffusion. One usually goes to b-value > 2000 s mm^−2^ for sensitivity to kurtosis, etc. That said, perfusion is measurable at low b-values < 200 s mm^−2^ ^47,48^. Usually, the low b-values account for blood perfusion, and the high b-values account for the water diffusion^47–50^. To account for both, we used the 11 different b-values to accurately find the differences in perfusion and diffusion patterns between the non-rejection and acute rejection groups. Additionally, we integrated the decision from the 11 different b-values to get an accurate final decision. It is worth noting that this integration helped with handling any errors that might occur in one or two b-values due to chemical shifts or existing artifacts during the acquisition process.

To summarize, a deep learning-based CAD system for non-invasive assessment of renal transplant status using the fusion of the DW-MR derived markers and clinical biomarkers was developed. Specifically, this fusion process produced well-separated CNN ADC input maps that were used as transplant status discriminatory features, which in turn affected the individual as well as the global diagnostic accuracy of the proposed CAD system. These preliminary results demonstrated the potential of the CAD system as a reliable non-invasive renal transplant diagnostic tool. It is independent of the scanner type and/or imaging protocol that has been used in DW-MRI data collection and the geographical area where the data were collected. The developed technique has also proven that the processing time and complexity can be reduced by avoiding the complex segmentation and registration procedures without affecting the quality of the final diagnosis.

Currently, we are including both lower and higher b-values without sacrificing any of the 11 different b-values to help us gather all possible information that might, one day, lead to the development of an accurate non-invasive alternative diagnostic tool to the renal biopsy. However, we are planning to extend our study by performing a statistical analysis to determine the most informative b-values. Once we reach this point, we could sacrifice some of the b-values that are unsatisfactory informative, which in turn will reduce the DW-MRI acquisition time. Future progress includes the usage of a larger sample size collected at different transplant centers and/or different imaging systems and collection protocols, and the exploration of additional biomarkers, such as genomic information that will augment personalized data in the cohort.

## Data Availability

The datasets generated during and/or analyzed during the current study are available from the corresponding author on a reasonable request.
